# A211 INCREASED RATES OF PPI DEPRESCRIPTION OVER TIME IN PATIENTS WITH UPPER GI BLEEDS

**DOI:** 10.1093/jcag/gwad061.211

**Published:** 2024-02-14

**Authors:** K Kecskemeti, M Borgaonkar, J Mcgrath

**Affiliations:** Memorial University of Newfoundland, St. John's, , Canada; Memorial University of Newfoundland, St. John's, , Canada; Memorial University of Newfoundland, St. John's, , Canada

## Abstract

**Background:**

Proton Pump Inhibitors (PPIs) are commonly used for various indications, including prophylaxis of upper GI bleeding. We aimed to assess if there has been more PPI deprescription in recent years in patients treated for Upper GI Bleeds (UGIB).

**Aims:**

Our study aimed to assess the frequency of PPI deprescription in patients treated with endoscopic therapy for UGIBs. We hypothesized that there was a higher rate of PPI deprescription in recent years.

**Methods:**

This retrospective cohort study analyzed patients who received endoscopic treatment for UGIB between the years 2015-2022. All patients who were treated with endoscopic therapy for an UGIB from two academic GI practices were included. PPI deprescription was defined as a 50% dose reduction, frequency reduction or complete medication discontinuation in the past 3 months or longer in established PPI users. To assess trends, the cohort was divided into group 1 (2015-2018) and group 2 (2019-2022). Data were analysed using SPSS and chi-squared testing was used to compare categorical variables.

**Results:**

Data were collected from 301 UGIB treated endoscopically. The mean age was 66.4 ± 13.5 years with 64% males and 36% females. Patients from group 1 had a deprescription rate of 2% (2/101 cases). Patients from group 2 (2019-2022) had a deprescription rate of 16% (32/200 cases) (pampersand:003C0.001).

**Conclusions:**

There was a higher rate of PPI deprescription in patients treated for UGIB in the second time period. Reasons for the increased rate of PPI deprescription require further investigation.

Chi-Squared of PPI Deprescription for entire cohort

Fisher Exact test: P value ampersand:003C0.001

**Funding Agencies:**

None

**Table:** 

Chi Squared	Group 1	Group 2	Total
PPI Deprescribed	2	32	34
PPI Non-deprescribed	99	168	267
Total	101	200	301

